# Supplementary material for: Molecular Hybrids of Thiazolidinone: Bridging Redox Modulation and Cancer Therapy
Source: Int J Mol Sci. 2025 Jul 7;26(13):6529. doi: 10.3390/ijms26136529 (PMC12249957; doi:10.3390/ijms26136529)
Supplement: Supplementary file 1 [file ijms-26-06529-s001.zip › ijms-3748658-supplementary.pdf]

Supplementary data for:

Molecular Hybrids of Thiazolidinone: Bridging Redox Modulation and Cancer Therapy

Nourah A. Al Zahrani [1], Manal A. Alshabibi [2], Abrar A. Bakr [2], Fahad A. Almughem [2], Abdullah A. Alshehri [2], Huda A. Al-Ghamdi [1], Essam A. Tawfik [2,*] and Laila A. Damiati [3,*]

[1] Department of Chemistry, College of Science, University of Jeddah, Jeddah 23218, Saudi Arabia;
nalzahrani2@uj.edu.sa (N.A.A.Z.); halgamdi4@uj.edu.sa (H.A.A.-G.)
[2] Advanced Diagnostics and Therapeutics Institute, Health Sector, King Abdulaziz City for Science and
Technology (KACST), Riyadh 11442, Saudi Arabia; malshabibi@kacst.gov.sa (M.A.A.); aabakr@kacst.gov.sa (A.A.B.); falmughem@kacst.gov.sa (F.A.A.); abdualshehri@kacst.gov.sa (A.A.A.)
[3] Department of Biological Science, College of Science, University of Jeddah, Jeddah 23218, Saudi Arabia
* Correspondence: etawfik@kacst.gov.sa (E.A.T.); ladamiati@uj.edu.sa (L.A.D.)

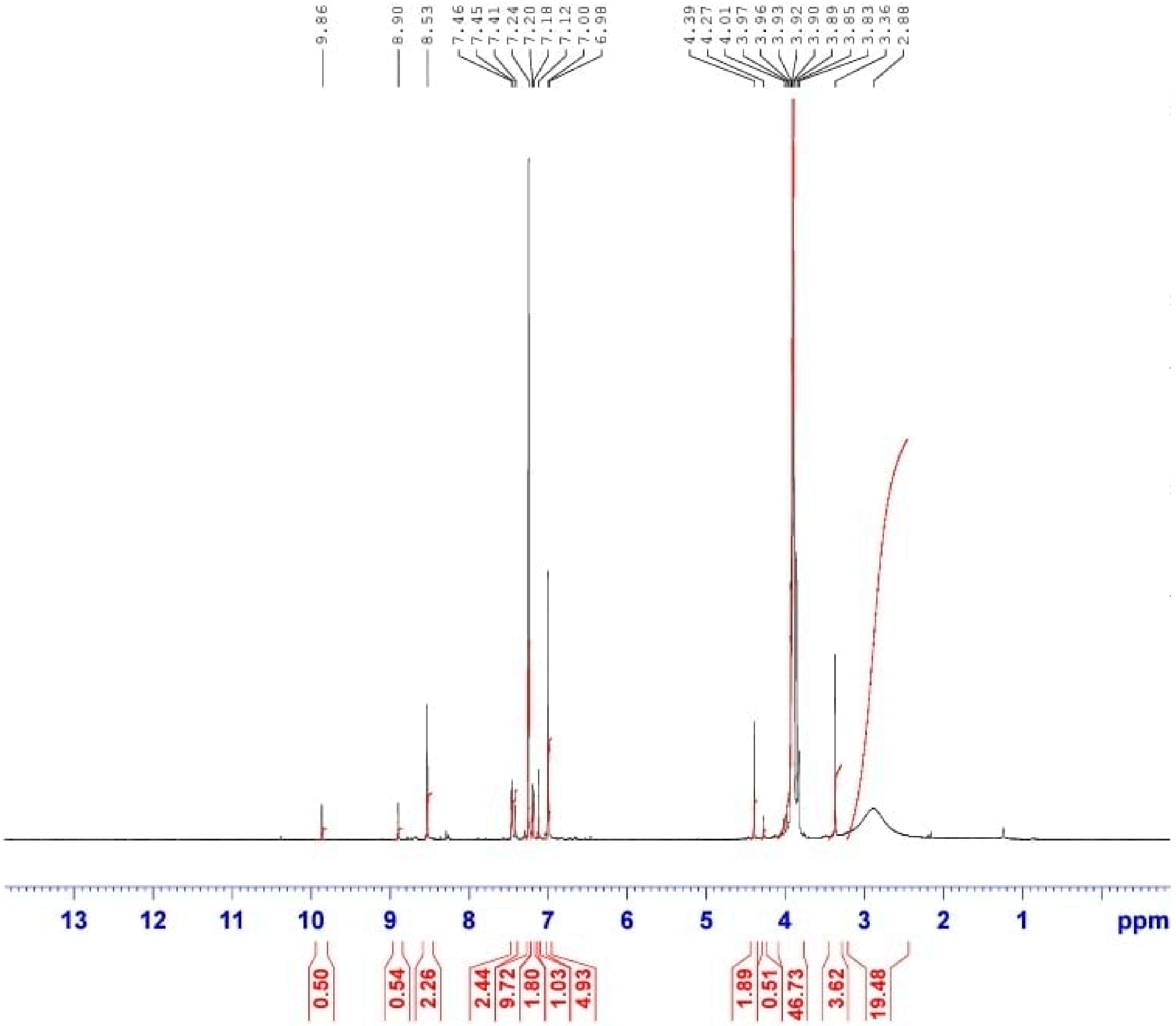

Supplementary Figure S1A. The ^{1}HNMR spectrum for thiazolidinone derivative 5.

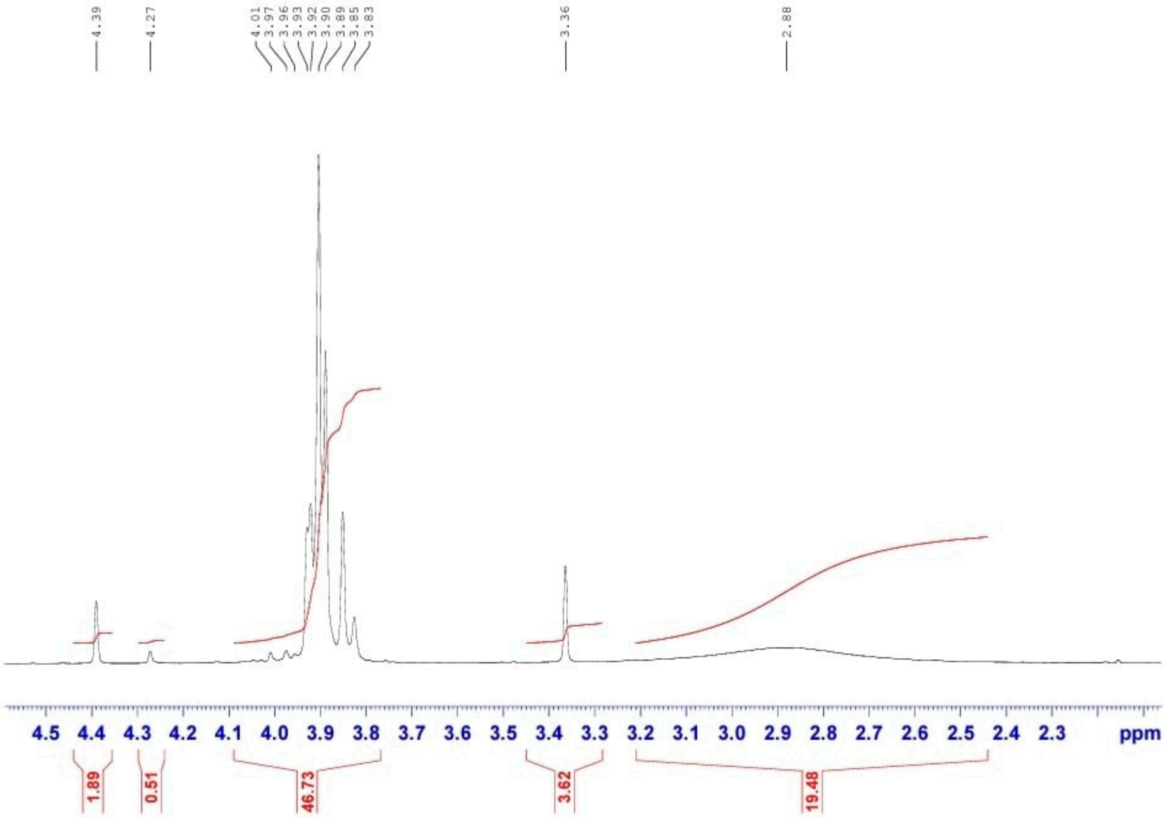

Supplementary Figure S1B. The ^{1}HNMR spectrum for thiazolidinone derivative 5

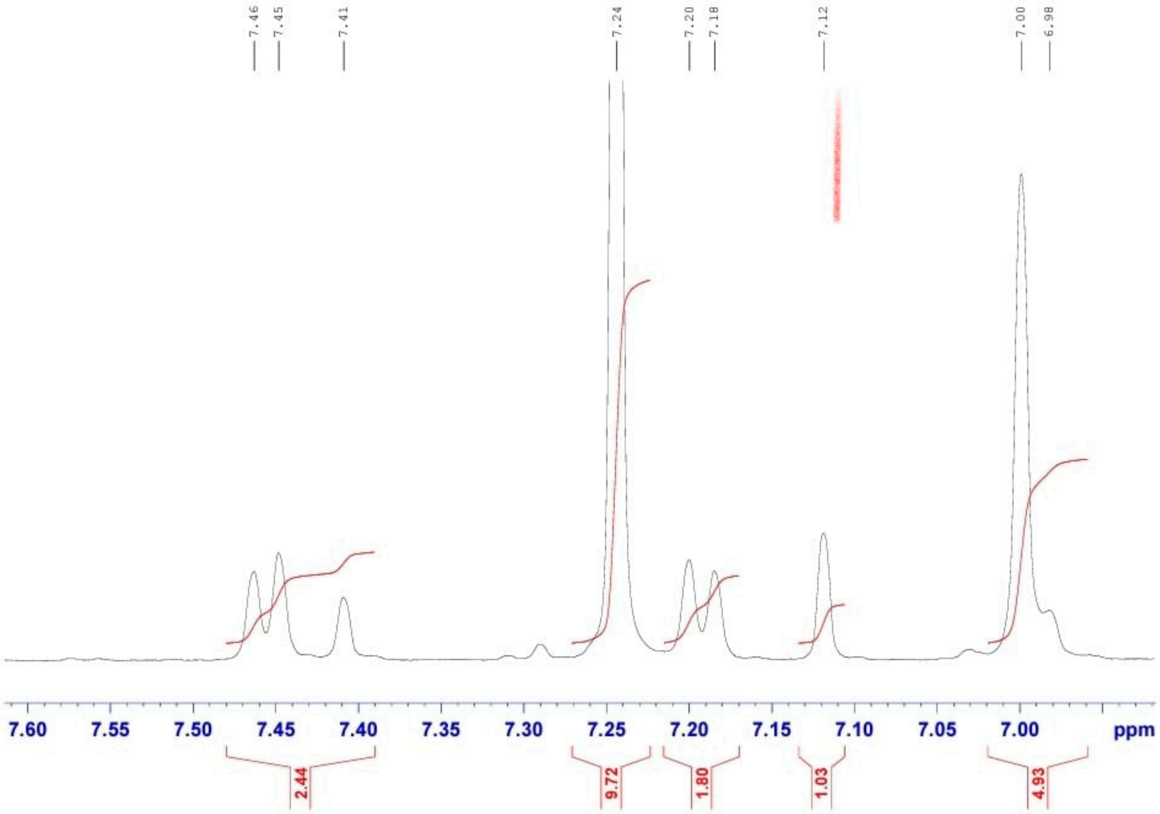

Supplementary Figure S1C. The ^{1}HNMR spectrum for thiazolidinone derivative 5

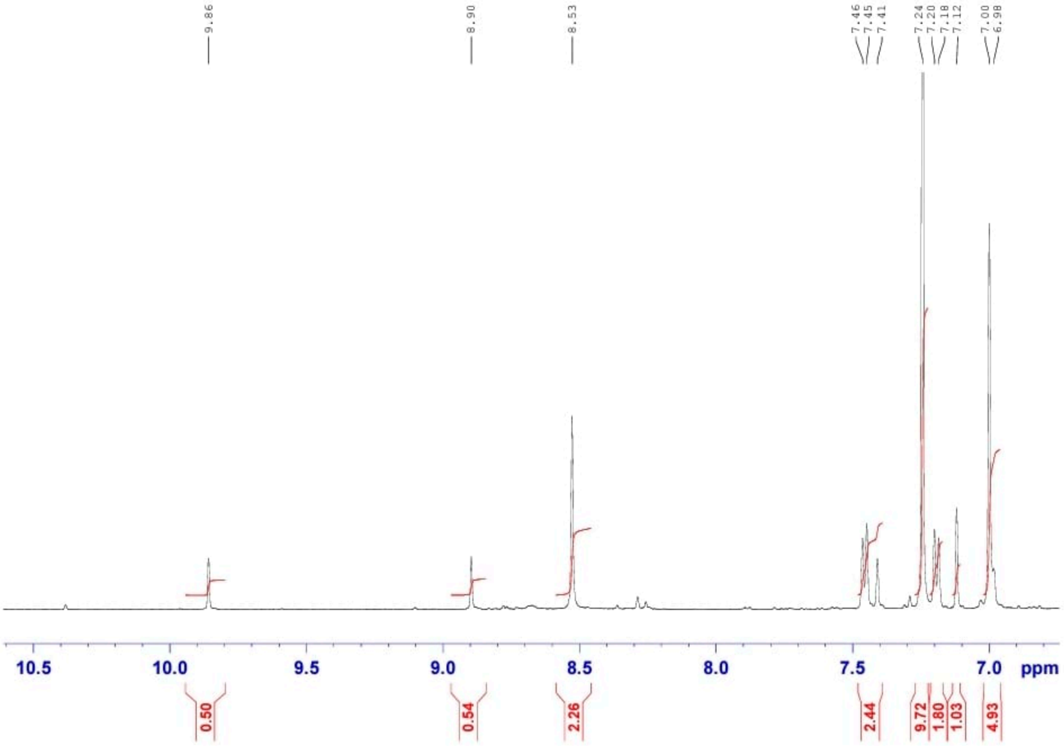

Supplementary Figure S1D. The ^{1}HNMR spectrum for thiazolidinone derivative 5.

The 1HNMR spectrum (A,B,C and D) showing the 1HNMR, δ in ppm :(500 MHz, CDCl3) δ 2.88 (s, CH_2, 2H), 3.85 (s, OCH_3, 3H), 3.90 (s, OCH_3, 3H), 3.93 (s, OCH_3, 3H), 3.97 (s, OCH_3, 3H), 4.39 (s, CH_2, 2H), 7.0 (s, Ar-H, 2H), 7.18 (d, J = 7Hz, Ar-H, 2H), 7.45 (d, J = 7 Hz, Ar-H, 2H), 8.53 (s, H–C=, 1H).

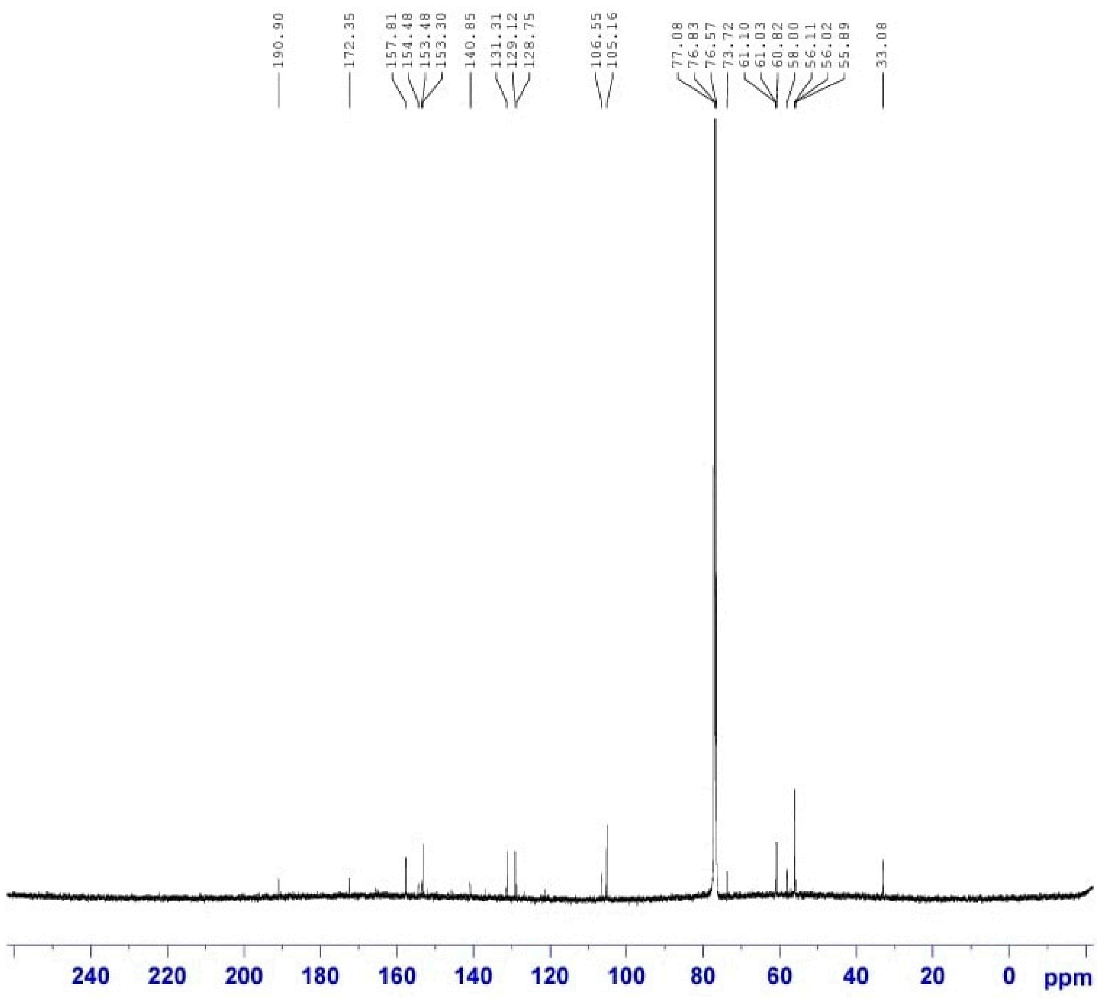

Supplementary Figure S2A. The ^{13}C NMR spectrum for thiazolidinone derivative 5.

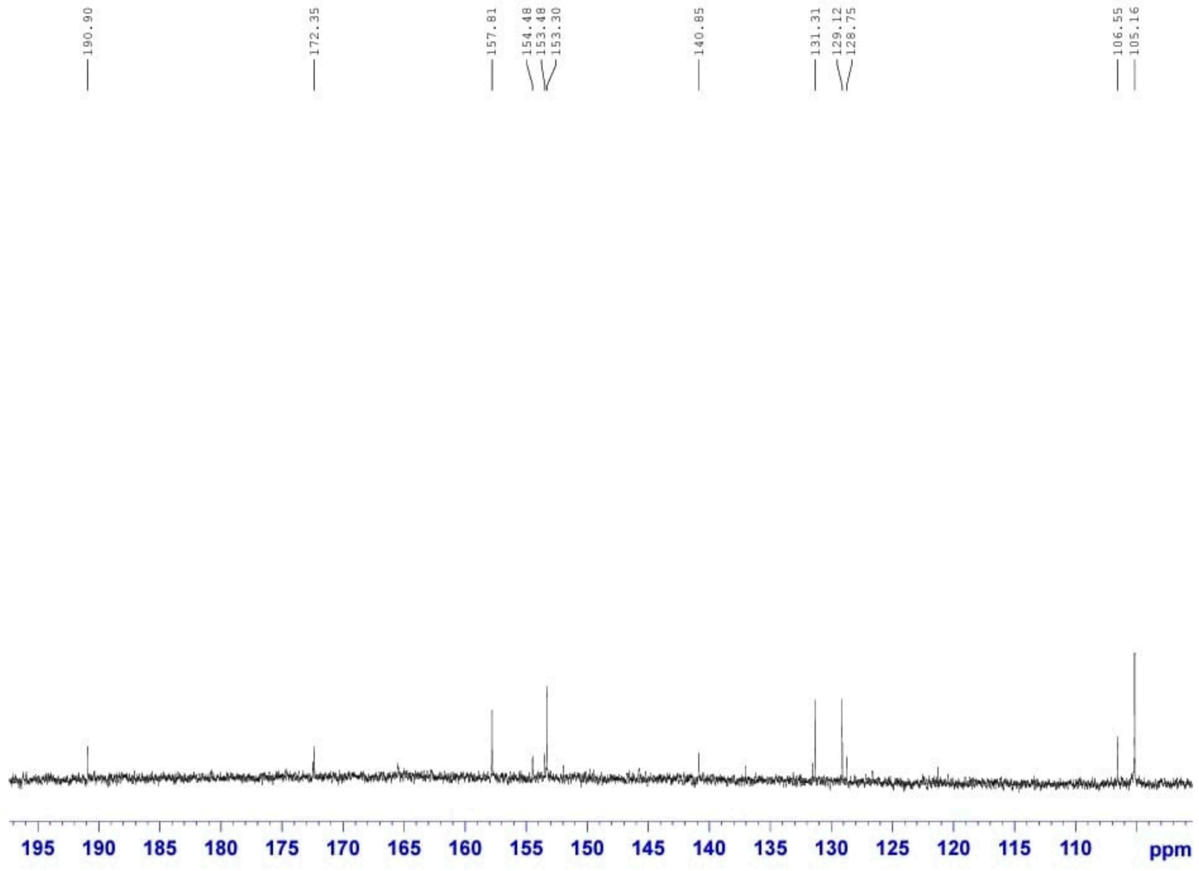

Supplementary Figure S2B. The ^{13}C NMR spectrum for thiazolidinone derivative 5.

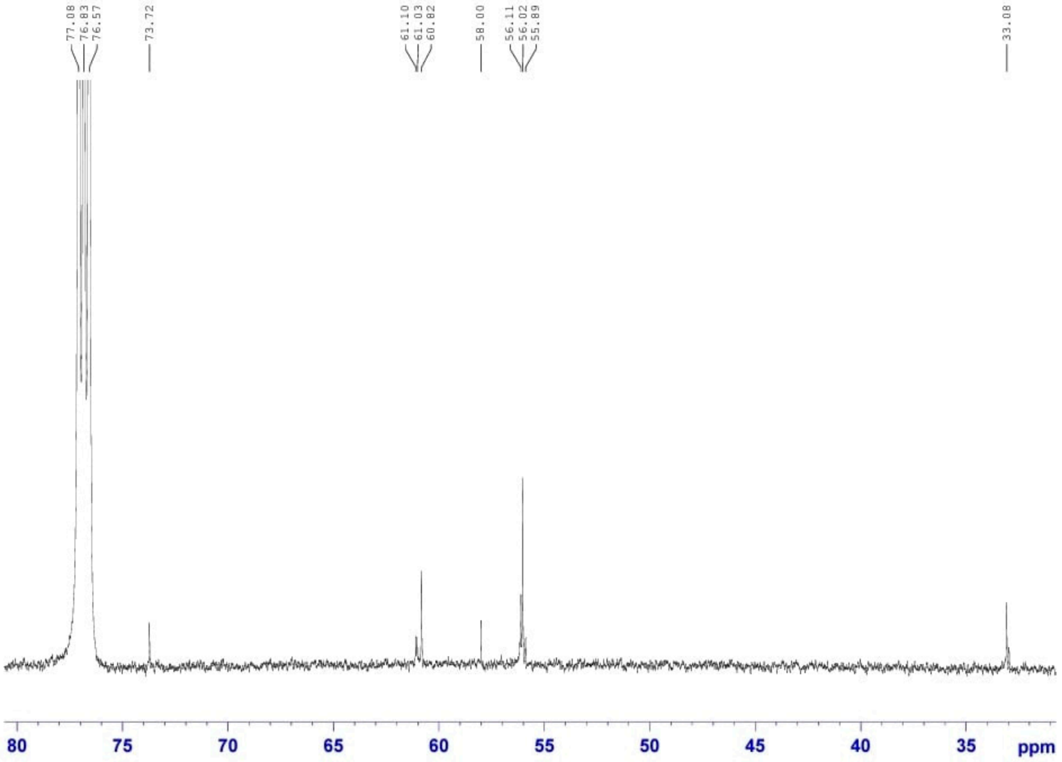

Supplementary Figure S2C. The ^{13}C NMR spectrum for thiazolidinone derivative 5.

The ^{13}C NMR spectrum (A, B and C) showing the ^{13}C NMR, δ in ppm :(500 MHz, CDCl3) δ 33.0, 56.0, 56.1, 58.0, 60.8, 105.1, 106.5, 128.7, 129.1, 131.1, 140.8, 153.3, 153.4, 154.4, 157.8, 172.3

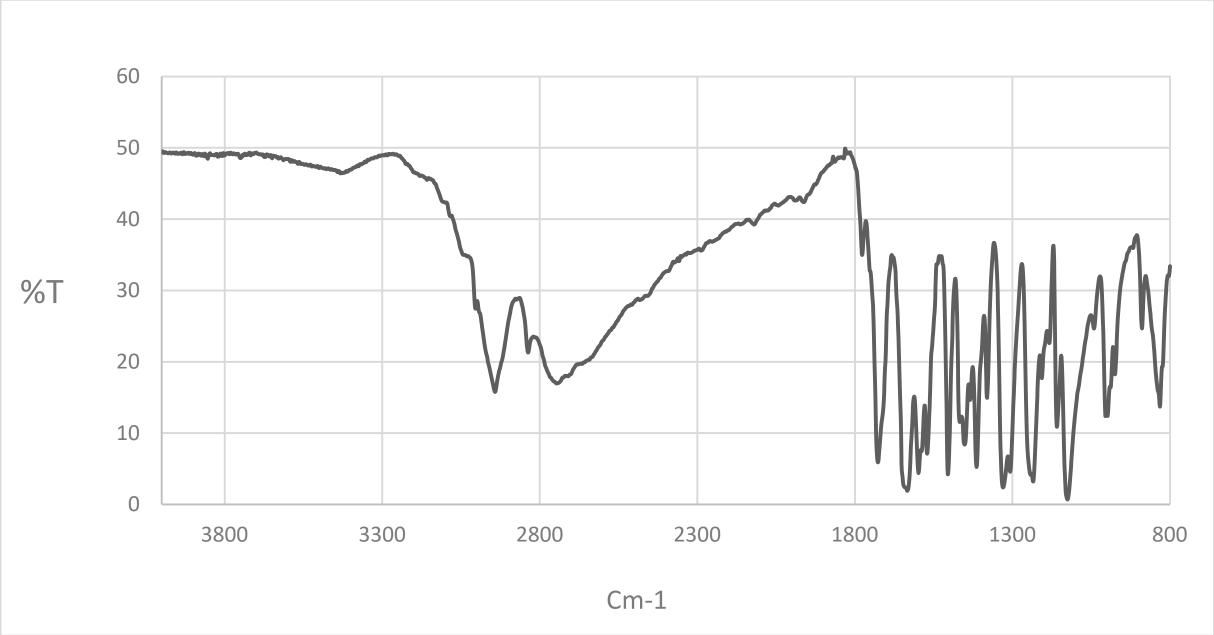

Supplementary Figure S3. The IR spectrum for thiazolidinone derivative 5 shows the distinctive peaks of IR cm^{-1}: 3031 (C–H sp^2), 2933(C–H sp^3), 1727 (C = O), 1634 (C = N), 1570, 1506 (C = C), 1234 (C–N), 1125 (C–O), 832 (C–H olefinic).

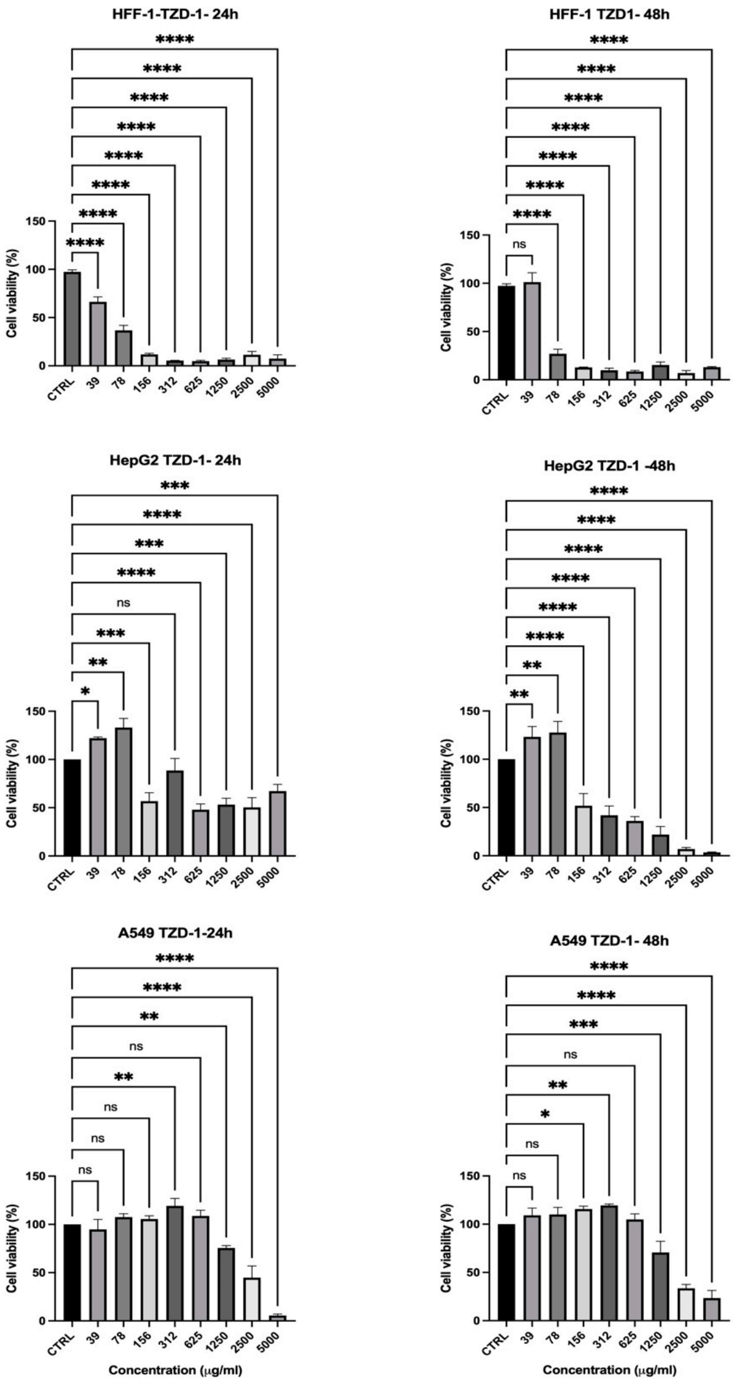

Supplementary Figure S4. Effect of **TZD-1** on cell viability of normal and cancer cell lines after 24 (left)-48 (right) h in culture. Cell viability was assessed using the MTS assay in: HFF-1, HepG2, and A549 after treatment with increasing concentrations of **TZD-1** (39–5000 μM). Viability was expressed as a percentage relative to the untreated cells (CTRL). Triton X-100 (0.1%) was used as a positive control for complete cell death. A dose-dependent decrease in viability was observed in cancer cell lines, while normal cells (HFF-1) showed higher resistance, indicating potential selectivity of **TZD-1** toward cancer cells. Data represents as mean ± SD, n=3. Statistical analysis was performed using One-Way ANOVA test, (*p<0.5, **p<0.01, ***p<0.001, **p<0.01, ****p<0.0001).

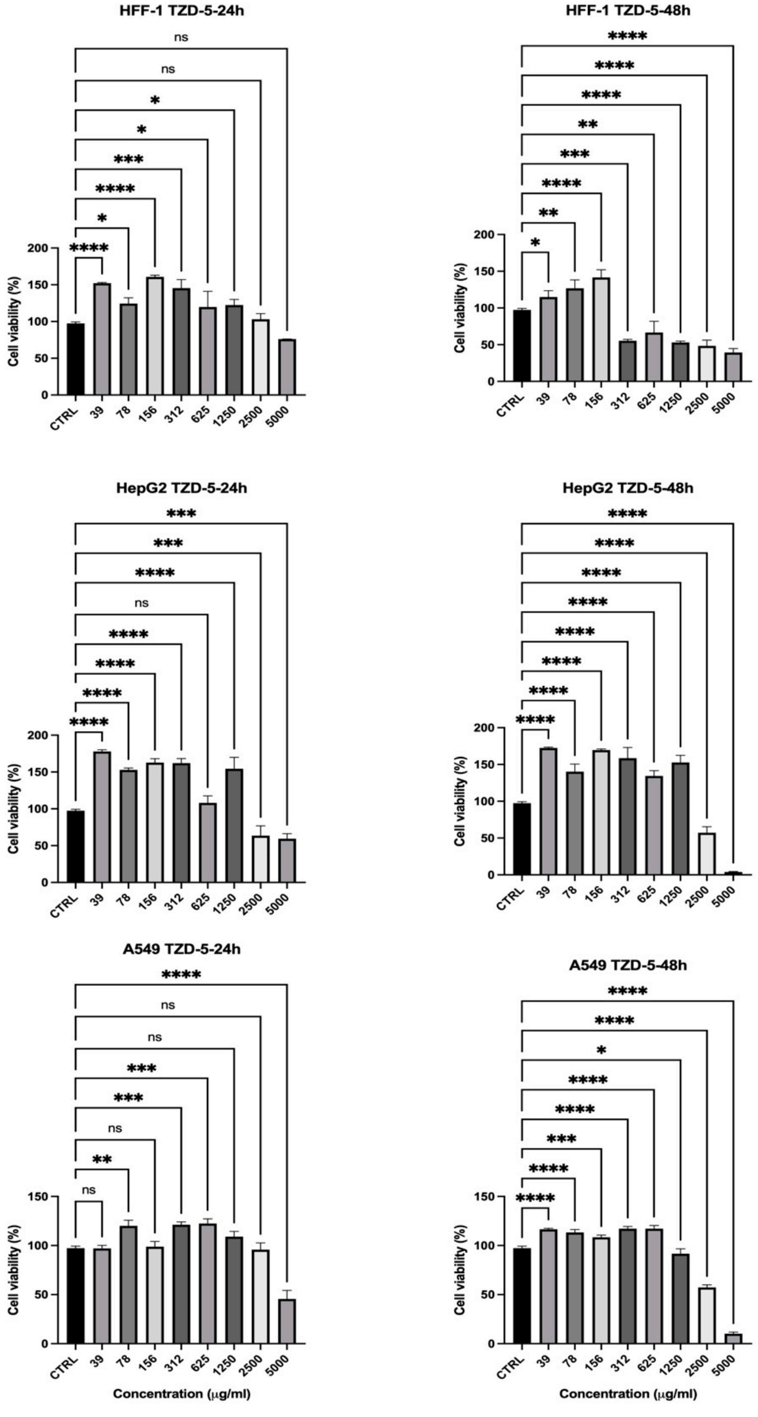

Supplementary Figure S5. Effect of **TZD-5** on cell viability of normal and cancer cell lines after 24 (left)-48 (right) h in culture. Viability was expressed as a percentage relative to the untreated cells (CTRL). Results showed a concentration- and time-dependent cytotoxic response, with HepG2 cells being relatively more sensitive compared to normal HFF-1 and A549 cancer cells, especially at higher concentrations. Data represents as mean ± SD, n=3. Statistical analysis was performed using One-Way ANOVA test, (*p<0.5, **p<0.01, ***p<0.001, **p<0.01, ****p<0.0001).

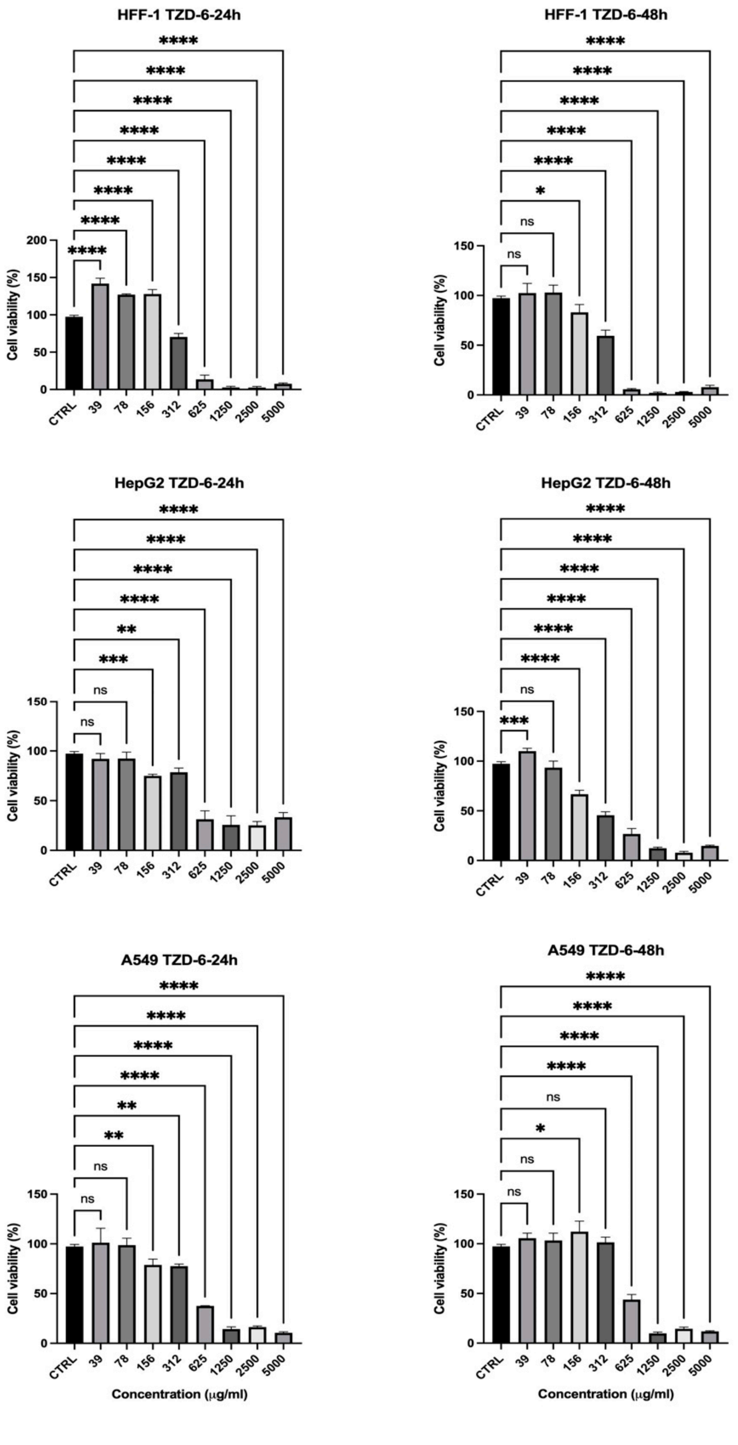

Supplementary Figure S6. Effect of **TZD-6** on cell viability of normal and cancer cell lines after 24 (left)-48 (right) h in culture. Viability is expressed as a percentage relative to the untreated cells (CTRL). The results highlighted that the **TZD-6** exhibits strong concentration- and time-dependent cytotoxicity against all tested cell lines, with significant reductions in viability observed at higher concentrations, showing limited selectivity between cancerous and normal cells. Data represents as mean ± SD, n=3. Statistical

analysis was performed using One-Way ANOVA test, (*p<0.5, **p<0.01, ***p<0.001, **p<0.01, ****p<0.0001).

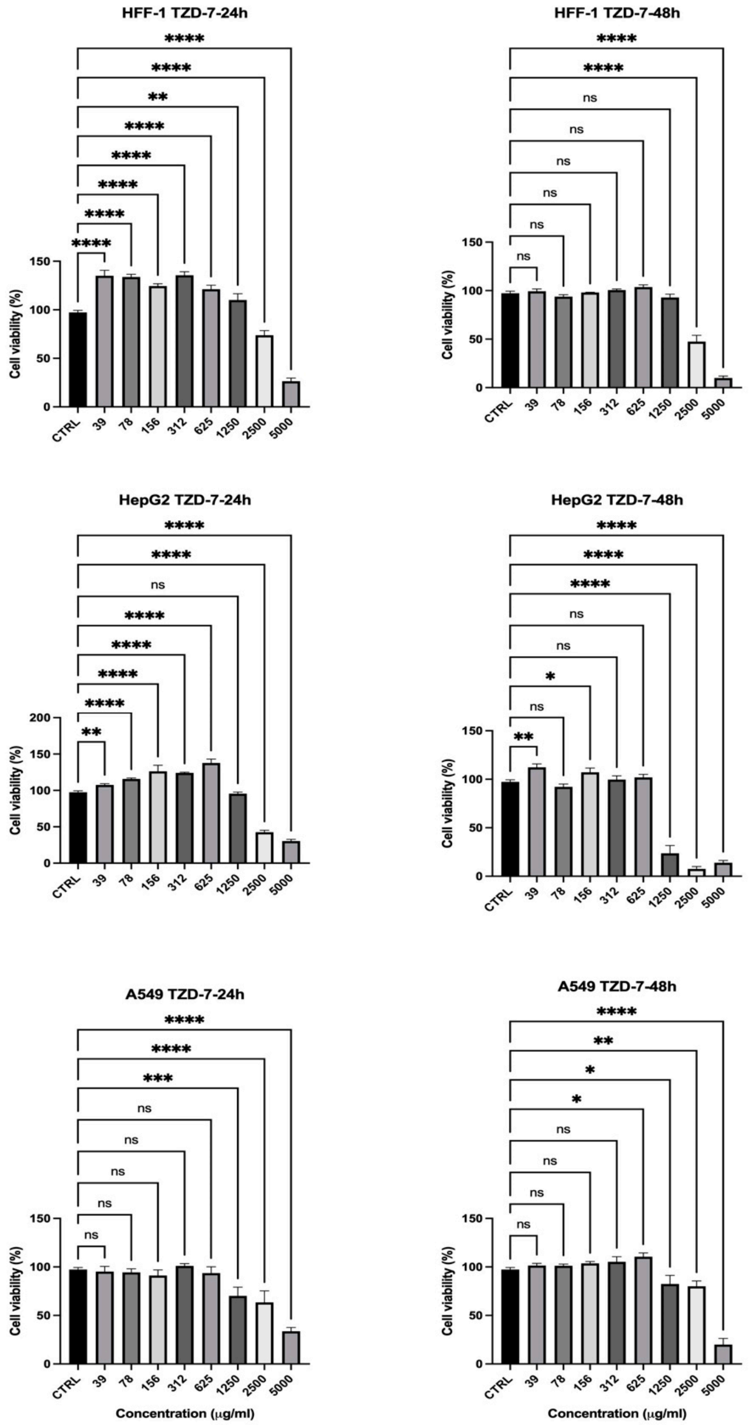

Supplementary Figure S7. Effect of **TZD-7** on cell viability of normal and cancer cell lines after 24 (left)-48 (right) h in culture. Viability is expressed as a percentage relative to the untreated cells (CTRL). **TZD-7** demonstrated selective cytotoxicity, effectively reducing the viability of cancer cells (HepG2 and A549), while exerting minimal effects on normal HFF-1 cells at similar concentrations, particularly after 48h of

exposure. Data represents as mean ± SD, n=3. Statistical analysis was performed using One-Way ANOVA test, (*p<0.5, **p<0.01, ***p<0.001, **p<0.01, ****p<0.0001).
